# Effects of inhaled high-molecular weight hyaluronan in inflammatory airway disease

**DOI:** 10.1186/s12931-016-0442-4

**Published:** 2016-10-03

**Authors:** Adelaida Lamas, Jamie Marshburn, Vandy P. Stober, Scott H. Donaldson, Stavros Garantziotis

**Affiliations:** 1 Hospital Universitario Ramón y Cajal, Instituto Ramón y Cajal de Investigación Sanitaria (IRYCIS), Madrid, Spain; 2 Division of Intramural Research, National Institute of Environmental Health Sciences, Research Triangle Park, Durham, NC USA; 3 University of North Carolina at Chapel Hill, Chapel Hill, NC USA; 4 National Institute of Environmental Health Sciences, 111 TW Alexander Dr, Research Triangle Park, Durham, NC 27709 USA

**Keywords:** Cystic fibrosis, Hyaluronan, Airway inflammation, Induced sputum

## Abstract

Cystic fibrosis (CF) is a chronic inflammatory disease that is affecting thousands of patients worldwide. Adjuvant anti-inflammatory treatment is an important component of cystic fibrosis treatment, and has shown promise in preserving lung function and prolonging life expectancy. Inhaled high molecular weight hyaluronan (HMW-HA) is reported to improve tolerability of hypertonic saline and thus increase compliance, and has been approved in some European countries for use as an adjunct to hypertonic saline treatment in cystic fibrosis. However, there are theoretical concerns that HMW-HA breakdown products may be pro-inflammatory. In this clinical pilot study we show that sputum cytokines in CF patients receiving HMW-HA are not increased, and therefore HMW-HA does not appear to adversely affect inflammatory status in CF airways.

## Introduction

Dear Editor,

Cystic fibrosis is a chronic inflammatory disease that is affecting thousands of patients worldwide. Although specific therapies like ivacaftor and lumacaftor/ivacaftor have been approved, they have high costs and are not indicated for all CF patients. Adjuvant anti-inflammatory treatment is an important component of cystic fibrosis treatment, and has shown promise in preserving lung function and prolonging life expectancy [1].

Airway inflammation generates short-fragment HA (sHA, MW 0.1-0.3 × 10^6^ Da) via degradation of structural high molecular hyaluronan (HMW-HA, MW > 10^6^ Da) or de novo expression, and further promotes inflammation and airway hyperresponsiveness. HMW-HA antagonizes sHA effects and has been studied therapeutically in animal models of inflammatory airway disease, such as asthma [2] and cystic fibrosis [3]. In addition, HMW-HA has been recently approved in some European countries for use in upper airway disease, and as an adjunct to hypertonic saline treatment in cystic fibrosis. Inhaled HMW-HA is reported to improve tolerability of hypertonic saline and thus increase compliance [4, 5]. Because HMW-HA ameliorates airway inflammation and hyperresponsiveness, its use in inflammatory lung disease has been advocated. However, a major concern clouding the therapeutic use of inhaled HMW-HA in inflammatory airway disease is that it may itself be degraded to sHA and thus contribute to inflammation. We, therefore, evaluated how chronic use of inhaled HMW-HA affects inflammatory cytokines in induced sputum of cystic fibrosis patients.

## Methods

We compared patients who were converted from treatment with hypertonic saline (HTS) to HTS with 0.1 % HMW-HA (MW 0.3-0.5 × 10^6^ Da, brand name Hyaneb®) because they could not tolerate the HTS solution. We performed two comparisons: 1) paired analysis in repeat sputa from patients before, and 4–6 weeks after conversion from HTS to HTS + HMW-HA; and 2) comparison of one group of patients on HTS with another group on HTS + HMW-HA. Patients were children and young adults with CF (11 male and 2 female, age (average ± SD) 15.6 ± 4.7) (Table 1).

**Table 1:** Lung function and clinical characteristics of patients transitioned from HTS to HTS_HMW-HA. Lung function values are expressed as % predicted

Patient	FEV1/FEV				P. aerug.						
	pre	post	gender	age	pre	post	inh Abx	AZT	BD	inh CS	dorn α
1	104	103	m	9	+	-	TOBI	+	+	+	+
2	101	104	m	19	-	-	colistin	+	+	+	-
3	95	85	f	13	-	-	TOBI	+	-	-	-
4	96	94	m	19	+	+	TOBI, colistin	+	+	+	-
5	113	113	m	18	-	-	-	-	+	-	-
6	102	104	m	8	-	-	-	-	-	-	-
7	87	102	m	16	+	+	TOBI	-	-	-	+
8	69	76	m	23	+	+	TOBI	-	-	-	+
9	104	104	m	23	+	+	TOBI, aztreonam	+	-	-	+
10	97	97	m	12	-	-	-	-	+	+	+
11	97	105	m	14	-	-	colistin	+	-	-	-
12	97	95	f	14	-	-	colistin, aztreonam	+	+	+	+
13	83	80	m	15	-	-	-	-	+	+	-
avg	95.8	97.1		15.6							
SD	11.0	10.9		4.7							
p	0.57										

*Abbreviations*: *avg* average value, *SD* standard deviation, *TOBI* inhaled tobramycin, *Abx* antibiotics, *AZT* azithromycin, *BD* chronic bronchodilator treatment, *CS* corticosteroids, *p* significance value in Wilcoxon matched-pairs, signed-rank test

## Results

**Fig. 1:**
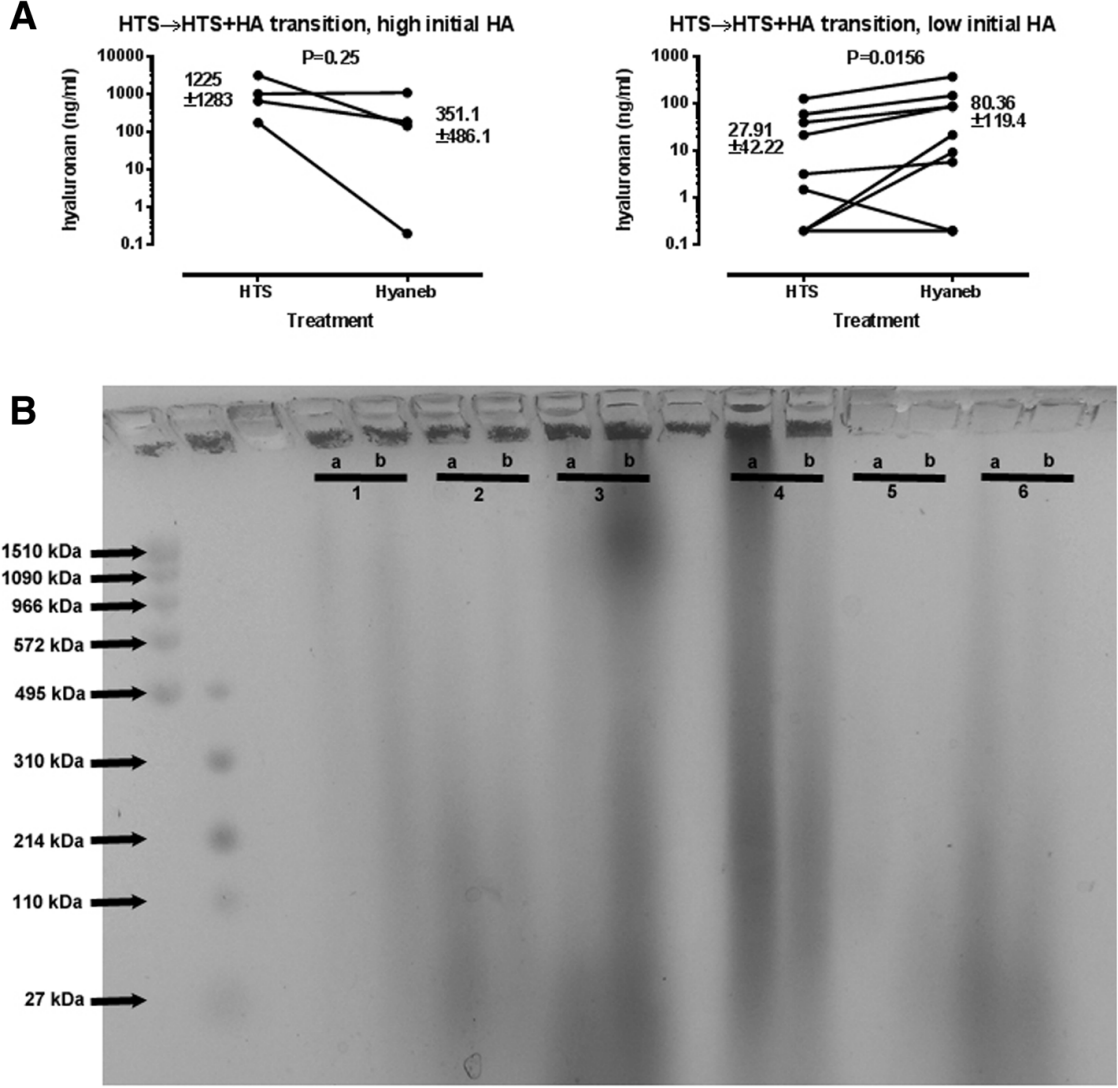
Levels of inflammation-related cytokines and chemokines in induced sputa of CF patients before and at least one month after induction of inhaled HMW-HA treatment (all patients were on hypertonic saline treatment throughout this period). Levels of HA in induced sputa of CF patients without inhaled HMW-HA treatment and at least one month after induction of inhaled HMW-HA treatment. Left panel depicts comparison of two separate groups of patients, on and off HMW-HA treatment, and right panel depicts HA levels in patients transitioned from HTS to HTS + HMW-HA treatment. **a** Levels of HA in induced sputa of CF patients before and at least one month after induction of inhaled HMW-HA treatment, divided according to initial HA levels prior to treatment initiation. **b** Agarose gel of HA sizes in induced sputa of CF patients before (lanes “a”) and at least one month after induction (lanes “b”) of inhaled HMW-HA treatment. Patients 1–3 had low initial HA levels, and patients 4–6 had high initial HA levels. In patients 1–3 there is apparent increase in HA, as well as shift of HA size towards larger molecular weights. In patients 4–6 there is evidence of decreased HA abundance but no apparent change in HA size

Lung function did not change before and after conversion in comparison 1 (FEV1/FVC pre 95.8 ± 11, FEV1/FVC post 97.1 ± 10.9), and was not different between groups in comparison 2. HMW-HA treatment did not increase induced sputum cytokine levels or HA levels in a significant manner (in paired comparisons average ± SD, pre vs post: IL-1β: 369 ± 794 vs 511 ± 1093 pg/ml; IL-6: 1.003 ± 1.775 vs 1.528 ± 1.968 ng/ml; IL-10: 1.140 ± 0.867 vs 1.225 ± 0.988 ng/ml; GM-CSF: 4.759 ± 4.452 vs 4.065 ± 3.342 ng/ml; TNF-α: 5.115 ± 6.477 vs 6.178 ± 10.76 ng/ml; not shown for between-groups comparison). There was large variability in sputum HA levels (range 0.2–2081 ng/ml for pre-HMW-HA, 0.2-1070 ng/ml for post-HMW-HA, mean and median 396 and 39.9 ng/ml for pre-HMW-HA, 163.7 and 85.3 ng/ml for post-HMW-HA). Interestingly, in patients with low initial HA levels in induced sputum, HTS + HMW-HA treatment led to a statistically significant increase in sputum HA levels. On the contrary, in patients with initially high sputum HA levels, initiation of inhaled HMW-HA led to no further increase, and perhaps a trend towards decreased HA levels. Initiation of HMW-HA treatment was reflected in an increase of HMW-HA in induced sputum (Fig. 1).

## Discussion

There are several potential reasons explaining why inhaled HMW-HA does not lead to increased inflammation in this population. First, even though there was apparent degradation of inhaled HMW-HA, there were still appreciable amounts of HMW-HA in induced sputum, suggesting that the degradation does not affect the entire inhaled dose. Thus, there may still be HMW-HA available to antagonize sHA. Second, sHA generated from the degradation of HMW-HA in the airway compartment may not reach interstitial immune cells in high enough concentrations to cause their activation. Lastly, sHA may not contribute significantly to inflammation in the context of pre-existing severe airway inflammation, although it is a proinflammatory agonist in the naïve lung. Interestingly, 100–150 kDa HA fragments (i.e. sHA) appear to be beneficial in the treatment of tobacco-induced airway disease, [6, 7] whereas they induce airway inflammation when given to naïve mice [8]. Thus, the theoretical concern about sHA aggravating airway disease may not be relevant in the real-world setting of pre-existing airway inflammation such as in cystic fibrosis or other chronic inflammatory diseases.

Although there was no apparent harm from inhaled HMW-HA in this population, there was also no apparent benefit in lung function or inflammatory cytokine expression. This particular formulation of inhaled HMW-HA is used to improve tolerability of HTS in CF [4, 9], not as an anti-inflammatory agent; nevertheless, HMW-HA has been previously shown to improve lung function in asthma and COPD [10–12] and inflammation in an animal model of CF [3]. Inhaled glycosaminoglycan formulations may have a role in airway disease. Recently, an exciting study reported significant improvements from inhaled heparin in patients with severe COPD. Inhaled HA has also been used successfully in COPD [6, 7, 13–15], and two clinical trials (NCT00993707 and NCT02674880) are currently underway evaluating efficacy of inhaled HA in COPD. However, HA effects in asthma and COPD may not parallel CF, where inflammation is more pronounced, and may overwhelm HMW-HA effects; furthermore, there are higher concentrations of sHA in CF [16] compared to asthma [17] which may antagonize HMW-HA; the thickness and tenacity of CF mucus may prevent HA from penetrating sufficiently into the airway cells layer; finally, lung function changes and inflammatory changes in CF have different kinetics than the dynamic airway obstruction in asthma, and thus the observation period in this study may not have been sufficiently long to detect changes. It is worth noting, that heparin demonstrated in vitro mucolytic effects in CF sputum [18], but inhaled heparin did not show significant improvement in lung function in a pilot study of CF patients [19]. Furthermore, some mechanisms of action of HA, such as inhibition of elastases and matrix breakdown [13, 14, 20] are relevant in COPD but not in CF. In aggregate, we believe that glycosaminoglycans like heparin and HA are likely to be effective in COPD and asthma, but that prolonged dosing, or different formulations may be needed for CF patients.

An interesting finding of our study involves the dichotomous effect of inhaled HMW-HA on HA induced sputum levels depending on initial concentration. In patients with low pre-treatment HA concentrations (defined as concentration < 100 ng/ml), initiation of treatment with HMW-HA led to an increase in measured levels. This may be reflecting the addition of inhaled HMW-HA to the airway fluid, and would suggest that inhaled HMW-HA does contribute to the HA pool in the airways. Given that HA is extremely hydrophilic, such increase may help hydrate the airways and contribute to bacterial clearance and improved ciliary function. More intriguing is the effect of inhaled HMW-HA when patients had initially high endogenous HA levels (>100 ng/ml): in this case there is an apparent decrease in sputum HA, which is evident both via ELISA and gel electrophoresis assays. It is possible that exogenous HMW-HA exerts a negative feedback effect in sHA generation, thus indirectly improving HA homeostasis towards less sHA and more HMW-HA.

## Conclusions

In summary, our results suggest that inhaled HMW-HA therapy does not exacerbate inflammation in airway disease, and may improve the HA homeostasis in inflamed lungs. Thus, inhaled HMW-HA merits investigation as an adjunct treatment for inflammatory airway disease.

## Abbreviations

Abx: Antibiotics
avg: Average value
AZT: Azithromycin
BD: Chronic bronchodilator treatment
CF: Cystic fibrosis
CS: Corticosteroids
FEV1: Forced expiratory volume in 1 s
FVC: Forced vital capacity
GM-CSF: Granulocyte-macrophage colony stimulating factor
HA: Hyaluronic acid, hyaluronan
HMW-HA: High molecular weight hyaluronan
HTS: Hypertonic saline
IL: Interleukin
SD: Standard deviation
TNF: Tumor necrosis factor
TOBI: Inhaled tobramycin
